# The genome sequence of Erhard’s wall lizard, *Podarcis erhardii *(Bedriaga, 1882)

**DOI:** 10.12688/wellcomeopenres.24041.1

**Published:** 2025-04-23

**Authors:** Nathalie Feiner, Tobias Uller, Kinsey M. Brock, Panayiotis Pafilis, Joana Meier, Wellcome Sanger Institute Tree of Life Management, Samples and Laboratory team, Wellcome Sanger Institute Scientific Operations: Sequencing Operations, Wellcome Sanger Institute Tree of Life Core Informatics team, Tree of Life Core Informatics collective

**Affiliations:** 1 Max Planck Institute for Evolutionary Biology, Plön, Germany; 2 Department of Biology, Lund University, Lund, Skåne County, Sweden; 3 Department of Biology, San Diego State University, San Diego, California, USA; 4 Biodiversity Museum, San Diego State University, San Diego, California, USA; 5 Department of Biology, National and Kapodistrian University of Athens, Athens, Greece; 6 Museum of Zoology, National and Kapodistrian University of Athens, Athens, Greece; 7 Wellcome Sanger Institute, Hinxton, England, UK

**Keywords:** Podarcis erhardii, Erhard's wall lizard, genome sequence, chromosomal, Squamata

## Abstract

We present a genome assembly from a female specimen of *Podarcis erhardii* (Erhard's wall lizard; Chordata; Lepidosauria; Squamata; Lacertidae). The assembly contains two haplotypes with total lengths of 1,495.98 megabases and 1,477.75 megabases. Most of haplotype 1 (99.28%) is scaffolded into 20 chromosomal pseudomolecules, including the W and Z sex chromosomes. Haplotype 2 was assembled to scaffold level. The mitochondrial genome has also been assembled, with a length of 17.14 kilobases.

## Species taxonomy

Eukaryota; Opisthokonta; Metazoa; Eumetazoa; Bilateria; Deuterostomia; Chordata; Craniata; Vertebrata; Gnathostomata; Teleostomi; Euteleostomi; Sarcopterygii; Dipnotetrapodomorpha; Tetrapoda; Amniota; Sauropsida; Sauria; Lepidosauria; Squamata; Bifurcata; Unidentata; Episquamata; Laterata; Lacertibaenia; Lacertidae; Lacertinae; *Podarcis*; *Podarcis erhardii* (Bedriaga, 1882) (NCBI:txid140042)

## Background

**Figure 1.**
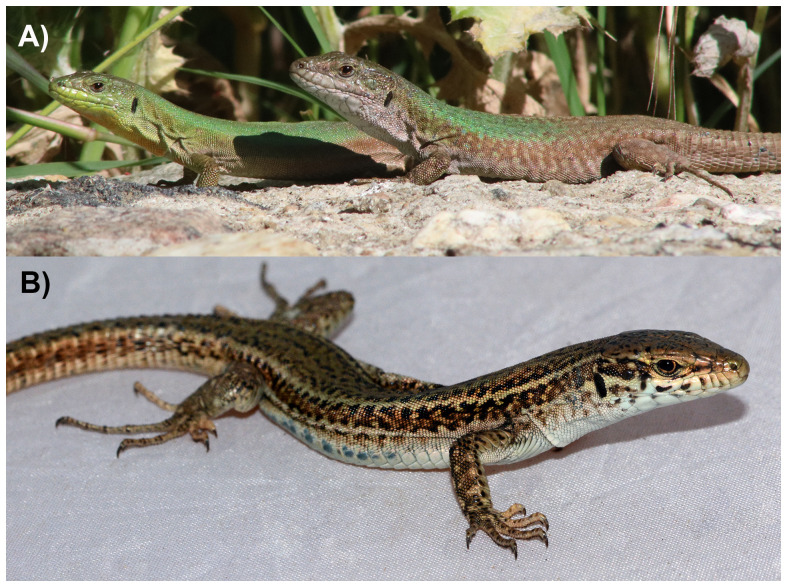
**A**) Female (left) and male (right) *Podarcis erhardii* photographed on the island of Andros, Greece. **B**) Female *P. erhardii* (rPodErh1) that was selected for genome sequencing upon capture on the island of Andros. Photographs by Kinsey Brock ( **A**) and John David Curlis ( **B**).

The Aegean wall lizard *Podarcis erhardii* (Bedriaga 1882; Figure 1), also commonly known as Erhard’s wall lizard, is named after the German naturalist Theodor Erhard. It is a diurnal lacertid lizard with a broad distribution in south-eastern Europe, occurring throughout Greece and northwards into the Balkan region. *P. erhardii* is classified as Least Concern (LC) by the IUCN ( Bowles & Crnobrnja-Isailović, 2024).

The Aegean wall lizard is one of 28 currently described species of the genus *Podarcis*. The species consists of several deeply divergent lineages that could justify a taxonomic revision ( Poulakakis *et al.*, 2003). *P. erhardii* is most closely related to the group of *P. cretensis*, *P. levendis*, *P. peloponnesiacus* and *P. thais*, which are also native to Greece ( Kiourtsoglou *et al.*, 2021; Yang *et al.*, 2021).

Typical of generalist species, Aegean wall lizards occur in a variety of habitats, including Mediterranean shrubland, grassland, sandy or rocky habitats as well as urban environments. The species is found from sea-level up to 2000 m elevation. Individuals can be active all year, but reproduction is generally restricted to spring and early summer. *P. erhardii* is oviparous and females lay between one and five eggs per clutch and multiple clutches per year ( Böhme, 1986). Meiri *et al.* (2020) reports an average clutch size of 2.53.

Populations of Aegean wall lizards are highly variable in colouration ( Escoriza, 2024) and body size ( Stadler *et al.*, 2023). The snout-to-vent length varies between 45 mm and 81 mm for adult males ( Valakos *et al.*, 2008) and 45 mm to 77 mm for adult females ( Stadler *et al.*, 2023). Colouration varies both within and between populations, with predominantly brown, grey and green hue and a variety of dorsal patterns. This species is one of several *Podarcis* that exhibit ventral colour polymorphism with yellow, orange and white morphs ( Brock *et al.*, 2020).

*Podarcis erhardii* belongs to the Balkan group of the genus *Podarcis*, which consists of 10 currently described species. Within this group, there is a reference genome available for *P. cretensis* ( Poulakakis *et al.*, 2024), and reference genomes for two other species ( *P. gaigeae* and *P. melisellensis*) have already been generated and will be published soon. Thus, the reference genome of *P. erhardii* will be a valuable resource, both for comparative studies above the species level, as well as studies addressing the evolutionary history, population structure, and phylogeography of *P. erhardii.*

The genome of the Erhard's wall lizard, *Podarcis erhardii*, was sequenced as part of the Darwin Tree of Life Project, a collaborative effort to sequence all named eukaryotic species in the Atlantic Archipelago of Britain and Ireland. Here we present a chromosomally complete genome sequence for *Podarcis erhardii*, based on a specimen from Andros ( Figure 1).

## Genome sequence report

### Sequencing data

The genome of a specimen of *Podarcis erhardii* ( Figure 1) was sequenced using Pacific Biosciences single-molecule HiFi long reads, generating 51.11 Gb (gigabases) from 4.93 million reads. GenomeScope analysis of the PacBio HiFi data estimated the haploid genome size at 1,440.47 Mb, with a heterozygosity of 0.59% and repeat content of 20.86%. These values provide an initial assessment of genome complexity and the challenges anticipated during assembly. Based on this estimated genome size, the sequencing data provided approximately 34.0x coverage of the genome. Chromosome conformation Hi-C sequencing produced 226.70 Gb from 1,501.35 million reads. Table 1 summarises the specimen and sequencing information.

**Table 1.** Specimen and sequencing data for *Podarcis erhardii*.

Project information			
**Study title**	Podarcis erhardii (Erhard's wall lizard)		
**Umbrella BioProject**	PRJEB78688		
**Species**	*Podarcis erhardii*		
**BioSpecimen**	SAMEA115336771		
**NCBI taxonomy ID**	140042		
Specimen information			
**Technology**	**ToLID**	**BioSample** **accession**	**Organism part**
**PacBio long read sequencing**	rPodErh1	SAMEA115336780	terminal body
**Hi-C sequencing**	rPodErh1	SAMEA115336780	terminal body
Sequencing information			
**Platform**	**Run accession**	**Read count**	**Base count (Gb)**
**Hi-C Illumina NovaSeq X**	ERR13474180	1.50e+09	226.7
**PacBio Revio**	ERR13485715	1.95e+06	19.48
**PacBio Revio**	ERR13485716	2.98e+06	31.63

### Assembly statistics

The genome was assembled into two haplotypes using Hi-C phasing. Haplotype 1 was curated to chromosome level, while haplotype 2 was assembled to scaffold level. The assembly was improved by manual curation, which corrected 73 misjoins or missing joins and removed 649 haplotypic duplications. These interventions decreased the scaffold count by 65.58%. The final assembly has a total length of 1,495.98 Mb in 325 scaffolds, with 836 gaps, and a scaffold N50 of 93.52 Mb ( Table 2).

**Table 2.** Genome assembly data for *Podarcis erhardii*.

Genome assembly	Haplotype 1	Haplotype 2
Assembly name	rPodErh1.hap1.1	rPodErh1.hap2.1
Assembly accession	GCA_964252035.1	GCA_964251985.1
Assembly level	chromosome	scaffold
Span (Mb)	1,495.98	1,477.75
Number of contigs	1,161	1,458
Number of scaffolds	325	647
Assembly metrics (benchmark)	Haplotype 1	Haplotype 2
Contig N50 length (≥ 1 Mb)	3.23 Mb	3.03 Mb
Scaffold N50 length *(= chromosome N50)*	93.52 Mb	93.57 Mb
Consensus quality (QV) (≥ 40)	63.9	61.5
*k*-mer completeness	86.58%	85.24%
Combined *k*-mer completeness (≥ 95%)	99.25%	
BUSCO * (S > 90%; D < 5%)	C:92.8%[S:91.0%,D:1.8%], F:0.8%,M:6.4%,n:7,480	C:92.3%[S:90.7%,D:1.6%], F:1.0%,M:6.7%,n:7,480
Percentage of assembly mapped to chromosomes (≥ 90%)	99.28%	*-*
Sex chromosomes (localised homologous pairs)	W and Z	*-*
Organelles (one complete allele)	Mitochondrial genome: 17.14 kb	*-*

* BUSCO scores based on the sauropsida_odb10 BUSCO set using version 5.5.0. C = complete [S = single copy, D = duplicated], F = fragmented, M = missing, n = number of orthologues in comparison.

**Figure 2.**
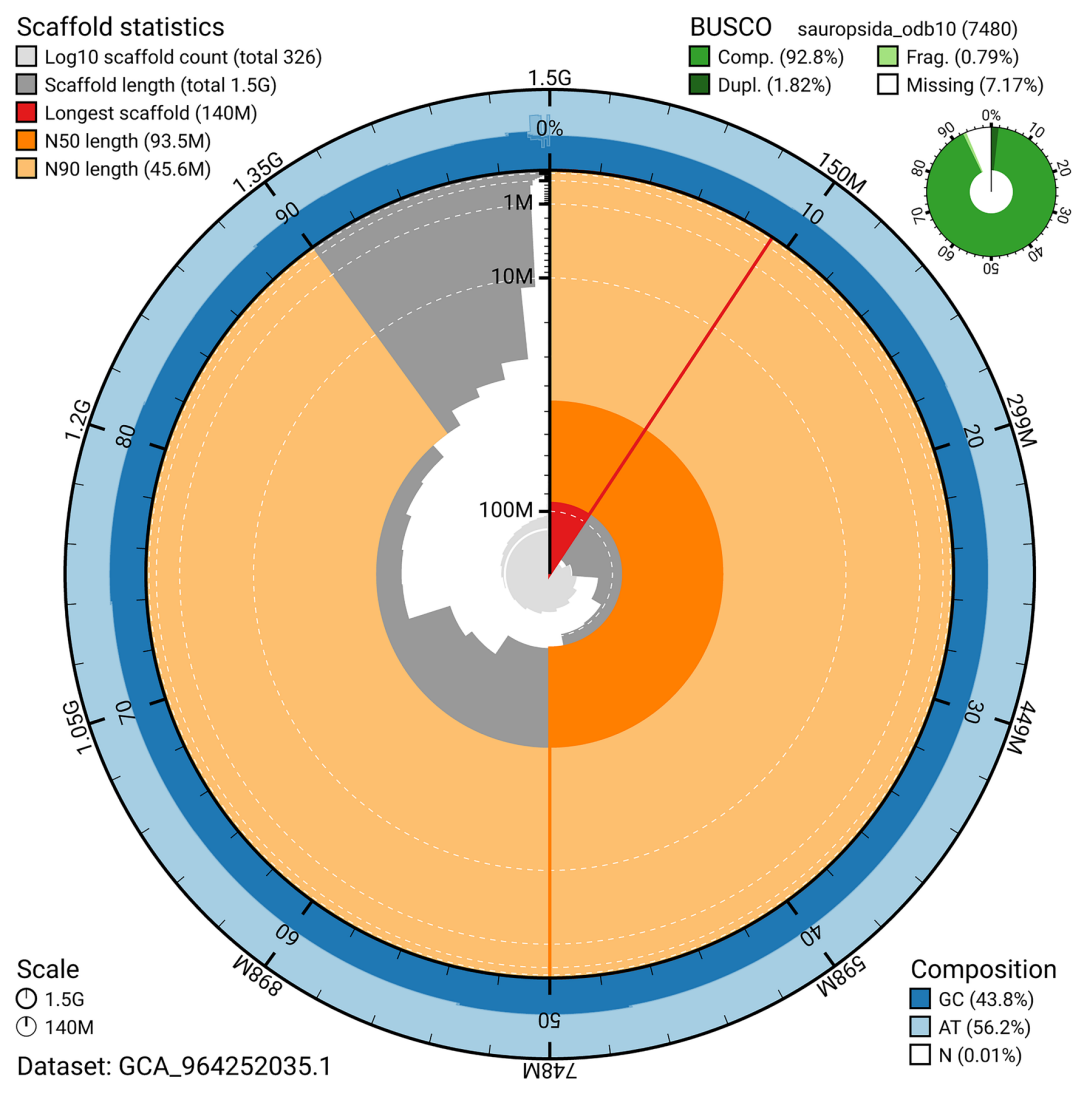
Genome assembly of *Podarcis erhardii*, rPodErh1.hap1.1: metrics. The BlobToolKit snail plot provides an overview of assembly metrics and BUSCO gene completeness. The circumference represents the length of the whole genome sequence, and the main plot is divided into 1,000 bins around the circumference. The outermost blue tracks display the distribution of GC, AT, and N percentages across the bins. Scaffolds are arranged clockwise from longest to shortest and are depicted in dark grey. The longest scaffold is indicated by the red arc, and the deeper orange and pale orange arcs represent the N50 and N90 lengths. A light grey spiral at the centre shows the cumulative scaffold count on a logarithmic scale. A summary of complete, fragmented, duplicated, and missing BUSCO genes in the sauropsida_odb10 set is presented at the top right. An interactive version of this figure is available at https://blobtoolkit.genomehubs.org/view/GCA_964252035.1/dataset/GCA_964252035.1/snail.

**Figure 3.**
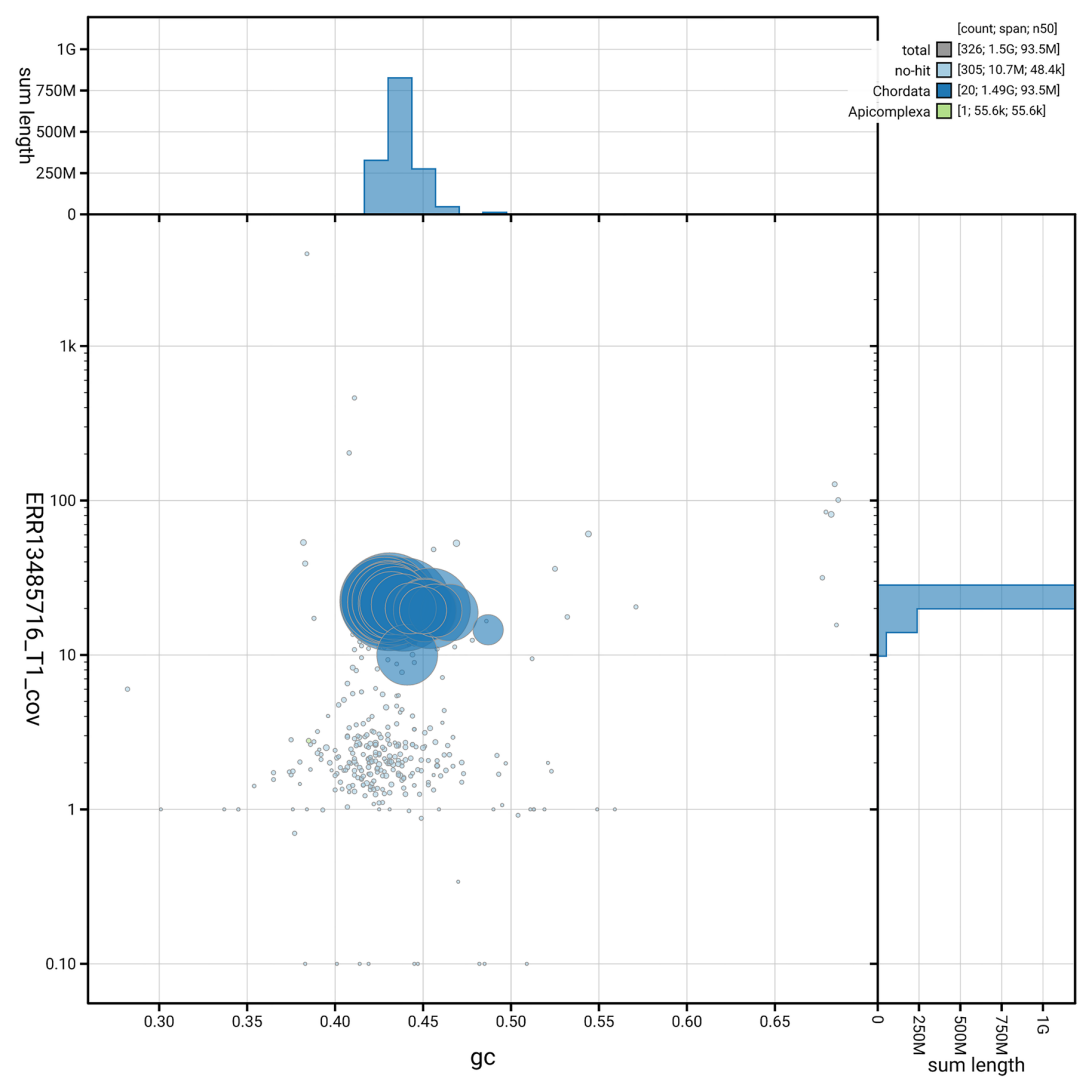
Genome assembly of *Podarcis erhardii*, rPodErh1.hap1.1: BlobToolKit GC-coverage plot. Blob plot showing sequence coverage (vertical axis) and GC content (horizontal axis). The circles represent scaffolds, with the size proportional to scaffold length and the colour representing phylum membership. The histograms along the axes display the total length of sequences distributed across different levels of coverage and GC content. An interactive version of this figure is available at https://blobtoolkit.genomehubs.org/view/GCA_964252035.1/dataset/GCA_964252035.1/blob.

**Figure 4.**
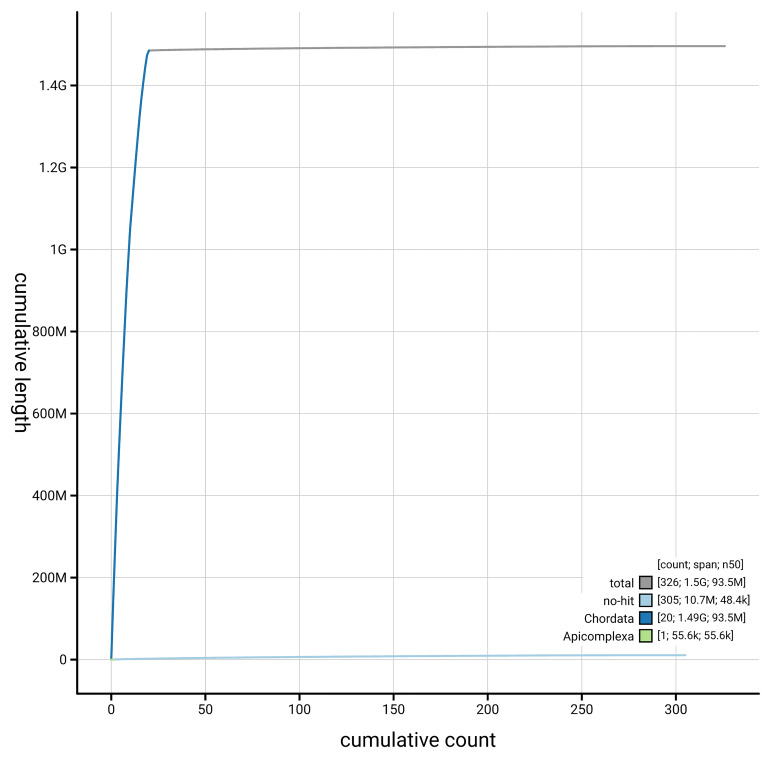
Genome assembly of *Podarcis erhardii*, rPodErh1.hap1.1: BlobToolKit cumulative sequence plot. The grey line shows cumulative length for all scaffolds. Coloured lines show cumulative lengths of scaffolds assigned to each phylum using the buscogenes taxrule. An interactive version of this figure is available at https://blobtoolkit.genomehubs.org/view/GCA_964252035.1/dataset/GCA_964252035.1/cumulative.

The snail plot in Figure 2 provides a summary of the assembly statistics, indicating the distribution of scaffold lengths and other assembly metrics. Figure 3 shows the distribution of scaffolds by GC proportion and coverage. Figure 4 presents a cumulative assembly plot, with separate curves representing different scaffold subsets assigned to various phyla, illustrating the completeness of the assembly.

**Figure 5.**
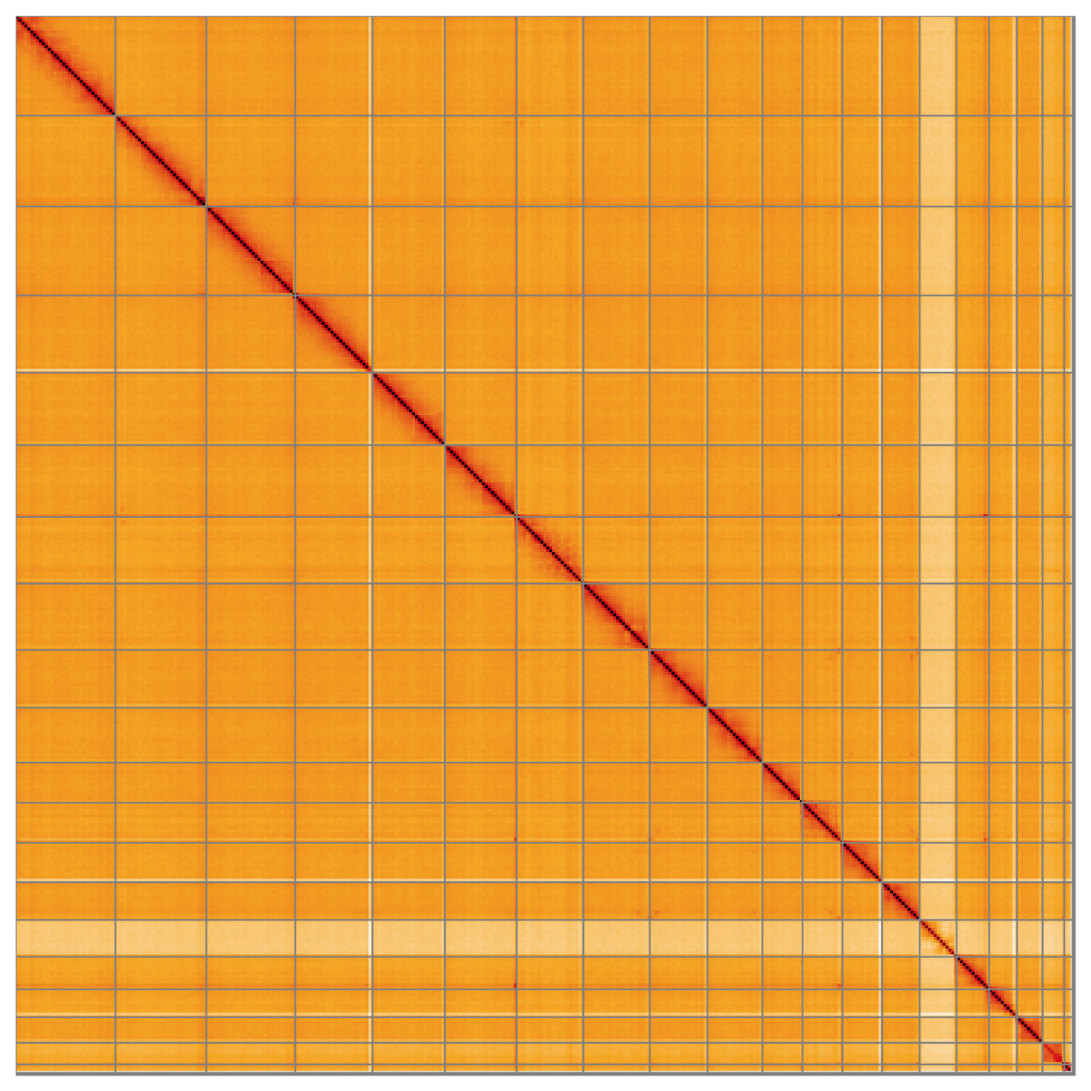
Genome assembly of *Podarcis erhardii*: Hi-C contact map of the rPodErh1.hap1.1 assembly, visualised using HiGlass. Chromosomes are shown in order of size from left to right and top to bottom. An interactive version of this figure may be viewed at https://genome-note-higlass.tol.sanger.ac.uk/l/?d=Nl3EYMFBTZudopXQwDabVA.

Most of the assembly sequence (99.28%) was assigned to 20 chromosomal-level scaffolds, representing 18 autosomes and the W and Z sex chromosomes. These chromosome-level scaffolds, confirmed by Hi-C data, are named according to size ( Figure 5; Table 3). During curation, the Z and W sex chromosomes were identified by ploidy and read coverage.

**Table 3.** Chromosomal pseudomolecules in the genome assembly of *Podarcis erhardii*, rPodErh1.

INSDC accession	Name	Length (Mb)	GC%
OZ176200.1	1	140.11	43
OZ176201.1	2	127.75	44
OZ176202.1	3	124.95	43
OZ176203.1	4	108.49	43
OZ176204.1	5	101.84	43
OZ176205.1	6	101.02	43.5
OZ176206.1	7	93.52	45.5
OZ176207.1	8	93.46	43
OZ176208.1	9	81.14	43
OZ176209.1	10	77.07	43.5
OZ176210.1	11	56.5	43
OZ176211.1	12	52.97	44
OZ176212.1	13	56.18	45
OZ176213.1	14	55.41	45
OZ176215.1	15	45.6	46.5
OZ176216.1	16	39.23	45.5
OZ176217.1	17	35.95	44.5
OZ176219.1	18	11.67	48.5
OZ176218.1	W	30.37	45
OZ176214.1	Z	51.98	44
OZ176220.1	MT	0.02	39.5

The mitochondrial genome was also assembled. This sequence is included as a contig in the multifasta file of the genome submission and as a standalone record.

### Assembly quality metrics

The estimated Quality Value (QV) and *k*-mer completeness metrics, along with BUSCO completeness scores, were calculated for each haplotype and the combined assembly. The QV reflects the base-level accuracy of the assembly, while *k*-mer completeness indicates the proportion of expected *k*-mers identified in the assembly. BUSCO scores provide a measure of completeness based on benchmarking universal single-copy orthologues.

For haplotype 1, the estimated QV is 63.9, and for haplotype 2, 61.5. When the two haplotypes are combined, the assembly achieves an estimated QV of 62.6. The *k*-mer recovery for haplotype 1 is 86.58%, and for haplotype 2 85.24%, while the combined haplotypes have a *k*-mer recovery of 99.25%. BUSCO 5.5.0 analysis using the sauropsida_odb10 reference set ( *n* = 7,480) identified 92.8% of the expected gene set (single = 91.0%, duplicated = 1.8%) for haplotype 1.

Table 2 provides assembly metric benchmarks adapted from Rhie *et al.* (2021) and the Earth BioGenome Project (EBP) Report on Assembly Standards September 2024. The haplotype 1 assembly achieves the EBP reference standard of **6.C.Q63**.

## Methods

### Sample acquisition

The specimen, an adult female *P. erhardii* lizard (specimen ID SAN25002444, ToLID rPodErh1) was collected on 2023-05-29 from a site on the island of Andros in the northern Cyclades (latitude: 37.85005159; longitude: 24.91615335). The specimen belongs to the subspecies *P. e. mykonensis*. The specimen was caught by noosing, standard morphometric measurements were taken, and the tip of the tail (ca. 2 cm) was collected and preserved in ethanol. The specimen was released again at the site of capture. Field work was conducted under the permit ID ΑΔΑ: Ψ9Ω04653Π8-ΓΩΓ.

### Nucleic acid extraction

The workflow for high molecular weight (HMW) DNA extraction at the Wellcome Sanger Institute (WSI) Tree of Life Core Laboratory includes a sequence of procedures: sample preparation and homogenisation, DNA extraction, fragmentation and purification. Detailed protocols are available on protocols.io ( Denton *et al.*, 2023b). The rPodErh1 sample was prepared for DNA extraction by weighing and dissecting it on dry ice ( Jay *et al.*, 2023). Tissue from the terminal body was homogenised using a PowerMasher II tissue disruptor ( Denton *et al.*, 2023a). HMW DNA was extracted using the Manual MagAttract v1 protocol ( Strickland *et al.*, 2023b). DNA was sheared into an average fragment size of 12–20 kb in a Megaruptor 3 system ( Todorovic *et al.*, 2023). Sheared DNA was purified by solid-phase reversible immobilisation, using AMPure PB beads to eliminate shorter fragments and concentrate the DNA ( Strickland *et al.*, 2023a). The concentration of the sheared and purified DNA was assessed using a Nanodrop spectrophotometer and Qubit Fluorometer using the Qubit dsDNA High Sensitivity Assay kit. Fragment size distribution was evaluated by running the sample on the FemtoPulse system.

### Hi-C sample preparation and crosslinking

Tissue from the terminal body of the rPodErh1 sample was processed for Hi-C sequencing at the WSI Scientific Operations core, using the Arima-HiC v2 kit. In brief, 20–50 mg of frozen tissue (stored at –80 °C) was fixed, and the DNA crosslinked using a TC buffer with 22% formaldehyde concentration. After crosslinking, the tissue was homogenised using the Diagnocine Power Masher-II and BioMasher-II tubes and pestles. Following the Arima-HiC v2 kit manufacturer's instructions, crosslinked DNA was digested using a restriction enzyme master mix. The 5’-overhangs were filled in and labelled with biotinylated nucleotides and proximally ligated. An overnight incubation was carried out for enzymes to digest remaining proteins and for crosslinks to reverse. A clean up was performed with SPRIselect beads prior to library preparation. Additionally, the biotinylation percentage was estimated using the Qubit Fluorometer v4.0 (Thermo Fisher Scientific) and Qubit HS Assay Kit and Arima-HiC v2 QC beads.

### Library preparation and sequencing

Library preparation and sequencing were performed at the WSI Scientific Operations core.

***PacBio HiFi***

At a minimum, samples were required to have an average fragment size exceeding 8 kb and a total mass over 400 ng to proceed to the low input SMRTbell Prep Kit 3.0 protocol (Pacific Biosciences, California, USA), depending on genome size and sequencing depth required. Libraries were prepared using the SMRTbell Prep Kit 3.0 (Pacific Biosciences, California, USA) as per the manufacturer's instructions. The kit includes the reagents required for end repair/A-tailing, adapter ligation, post-ligation SMRTbell bead cleanup, and nuclease treatment. Following the manufacturer’s instructions, size selection and clean up was carried out using diluted AMPure PB beads (Pacific Biosciences, California, USA). DNA concentration was quantified using the Qubit Fluorometer v4.0 (Thermo Fisher Scientific) with Qubit 1X dsDNA HS assay kit and the final library fragment size analysis was carried out using the Agilent Femto Pulse Automated Pulsed Field CE Instrument (Agilent Technologies) and gDNA 55kb BAC analysis kit.

Samples were sequenced on a Revio instrument (Pacific Biosciences, California, USA). Prepared libraries were normalised to 2 nM, and 15 μL was used for making complexes. Primers were annealed and polymerases were hybridised to create circularised complexes according to manufacturer’s instructions. The complexes were purified with the 1.2X clean up with SMRTbell beads. The purified complexes were then diluted to the Revio loading concentration (in the range 200–300 pM), and spiked with a Revio sequencing internal control. Samples were sequenced on Revio 25M SMRT cells (Pacific Biosciences, California, USA). The SMRT link software, a PacBio web-based end-to-end workflow manager, was used to set-up and monitor the run, as well as perform primary and secondary analysis of the data upon completion.

### Hi-C

For Hi-C library preparation, DNA was fragmented using the Covaris E220 sonicator (Covaris) and size selected using SPRISelect beads to 400 to 600 bp. The DNA was then enriched using the Arima-HiC v2 kit Enrichment beads. Using the NEBNext Ultra II DNA Library Prep Kit (New England Biolabs) for end repair, A-tailing, and adapter ligation. This uses a custom protocol which resembles the standard NEBNext Ultra II DNA Library Prep protocol but where library preparation occurs while DNA is bound to the Enrichment beads. For library amplification, 10 to 16 PCR cycles were required, determined by the sample biotinylation percentage. The Hi-C sequencing was performed using paired-end sequencing with a read length of 150 bp on an Illumina NovaSeq X instrument.

### Genome assembly, curation and evaluation

***Assembly***

Prior to assembly of the PacBio HiFi reads, a database of *k*-mer counts ( *k* = 31) was generated from the filtered reads using FastK. GenomeScope2 ( Ranallo-Benavidez *et al.*, 2020) was used to analyse the *k*-mer frequency distributions, providing estimates of genome size, heterozygosity, and repeat content.

The HiFi reads were assembled using Hifiasm in Hi-C phasing mode ( Cheng *et al.,* 2021; Cheng *et al.*, 2022), resulting in a pair of haplotype-resolved assemblies. The Hi-C reads were mapped to the primary contigs using bwa-mem2 ( Vasimuddin *et al.*, 2019). The contigs were further scaffolded using the provided Hi-C data ( Rao *et al.*, 2014) in YaHS ( Zhou *et al.*, 2023) using the --break option for handling potential misassemblies. The scaffolded assemblies were evaluated using Gfastats ( Formenti *et al.*, 2022), BUSCO ( Manni *et al.*, 2021) and MERQURY.FK ( Rhie *et al.*, 2020).

The mitochondrial genome was assembled using MitoHiFi ( Uliano-Silva *et al.*, 2023), which runs MitoFinder ( Allio *et al.*, 2020) and uses these annotations to select the final mitochondrial contig and to ensure the general quality of the sequence.

***Assembly curation***

The assembly was decontaminated using the Assembly Screen for Cobionts and Contaminants (ASCC) pipeline. Flat files and maps used in curation were generated via the TreeVal pipeline ( Pointon *et al.*, 2023). Manual curation was conducted primarily in PretextView ( Harry, 2022) and HiGlass ( Kerpedjiev *et al.*, 2018), with additional insights provided by JBrowse2 ( Diesh *et al.*, 2023). Scaffolds were visually inspected and corrected as described by Howe *et al*. (2021). Any identified contamination, missed joins, and mis-joins were amended, and duplicate sequences were tagged and removed. The curation process is documented at https://gitlab.com/wtsi-grit/rapid-curation.

***Assembly quality assessment***

The Merqury.FK tool ( Rhie *et al.*, 2020), run in a Singularity container ( Kurtzer *et al.*, 2017), was used to evaluate *k*-mer completeness and assembly quality for both haplotypes using the *k*-mer databases ( *k* = 31) computed prior to genome assembly. The analysis outputs included assembly QV scores and completeness statistics.

A Hi-C contact map was produced for the final version of the assembly. The Hi-C reads were aligned using bwa-mem2 ( Vasimuddin *et al.*, 2019) and the alignment files were combined using SAMtools ( Danecek *et al.*, 2021). The Hi-C alignments were converted into a contact map using BEDTools ( Quinlan & Hall, 2010) and the Cooler tool suite ( Abdennur & Mirny, 2020). The contact map was visualised in HiGlass ( Kerpedjiev *et al.*, 2018).

The blobtoolkit pipeline is a Nextflow ( Di Tommaso *et al.*, 2017) port of the previous Snakemake Blobtoolkit pipeline ( Challis *et al.*, 2020). It aligns the PacBio reads in SAMtools and minimap2 ( Li, 2018) and generates coverage tracks for regions of fixed size. In parallel, it queries the GoaT database ( Challis *et al.*, 2023) to identify all matching BUSCO lineages to run BUSCO ( Manni *et al.*, 2021). For the three domain-level BUSCO lineages, the pipeline aligns the BUSCO genes to the UniProt Reference Proteomes database ( Bateman *et al.*, 2023) with DIAMOND blastp ( Buchfink *et al.*, 2021). The genome is also divided into chunks according to the density of the BUSCO genes from the closest taxonomic lineage, and each chunk is aligned to the UniProt Reference Proteomes database using DIAMOND blastx. Genome sequences without a hit are chunked using seqtk and aligned to the NT database with blastn ( Altschul *et al.*, 1990). The blobtools suite combines all these outputs into a blobdir for visualisation.

The blobtoolkit pipeline was developed using nf-core tooling ( Ewels *et al.*, 2020) and MultiQC ( Ewels *et al.*, 2016), relying on the Conda package manager, the Bioconda initiative ( Grüning *et al.*, 2018), the Biocontainers infrastructure ( da Veiga Leprevost *et al.*, 2017), as well as the Docker ( Merkel, 2014) and Singularity ( Kurtzer *et al.*, 2017) containerisation solutions.

Table 4 contains a list of relevant software tool versions and sources.

**Table 4.** Software tools: versions and sources.

Software tool	Version	Source
BEDTools	2.30.0	https://github.com/arq5x/bedtools2
BLAST	2.14.0	ftp://ftp.ncbi.nlm.nih.gov/blast/executables/blast+/
BlobToolKit	4.3.9	https://github.com/blobtoolkit/blobtoolkit
BUSCO	5.5.0	https://gitlab.com/ezlab/busco
bwa-mem2	2.2.1	https://github.com/bwa-mem2/bwa-mem2
Cooler	0.8.11	https://github.com/open2c/cooler
DIAMOND	2.1.8	https://github.com/bbuchfink/diamond
fasta_windows	0.2.4	https://github.com/tolkit/fasta_windows
FastK	666652151335353eef2fcd58880bcef5bc2928e1	https://github.com/thegenemyers/FASTK
Gfastats	1.3.6	https://github.com/vgl-hub/gfastats
GoaT CLI	0.2.5	https://github.com/genomehubs/goat-cli
Hifiasm	0.19.8-r603	https://github.com/chhylp123/hifiasm
HiGlass	44086069ee7d4d3f6f3f0012569789ec138f42b84aa44357826c0b6753eb28de	https://github.com/higlass/higlass
MerquryFK	d00d98157618f4e8d1a9190026b19b471055b22e	https://github.com/thegenemyers/MERQURY.FK
Minimap2	2.24-r1122	https://github.com/lh3/minimap2
MitoHiFi	3	https://github.com/marcelauliano/MitoHiFi
MultiQC	1.14, 1.17, and 1.18	https://github.com/MultiQC/MultiQC
Nextflow	23.10.0	https://github.com/nextflow-io/nextflow
PretextView	0.2.5	https://github.com/sanger-tol/PretextView
samtools	1.19.2	https://github.com/samtools/samtools
sanger-tol/ascc	-	https://github.com/sanger-tol/ascc
sanger-tol/blobtoolkit	0.5.1	https://github.com/sanger-tol/blobtoolkit
Seqtk	1.3	https://github.com/lh3/seqtk
Singularity	3.9.0	https://github.com/sylabs/singularity
TreeVal	1.2.0	https://github.com/sanger-tol/treeval
YaHS	1.2a.2	https://github.com/c-zhou/yahs

### Wellcome Sanger Institute – Legal and Governance

The materials that have contributed to this genome note have been supplied by a Tree of Life collaborator. The Wellcome Sanger Institute employs a process whereby due diligence is carried out proportionate to the nature of the materials themselves, and the circumstances under which they have been/are to be collected and provided for use. The purpose of this is to address and mitigate any potential legal and/or ethical implications of receipt and use of the materials as part of the research project, and to ensure that in doing so we align with best practice wherever possible.

The overarching areas of consideration are:

  •    Ethical review of provenance and sourcing of the material

  •    Legality of collection, transfer and use (national and international)

Each transfer of samples is undertaken according to a Research Collaboration Agreement or Material Transfer Agreement entered into by the Tree of Life collaborator, Genome Research Limited (operating as the Wellcome Sanger Institute) and in some circumstances other Tree of Life collaborators.

## Data Availability

European Nucleotide Archive: Podarcis erhardii (Erhard's wall lizard). Accession number PRJEB78688; https://identifiers.org/ena.embl/PRJEB78688. The genome sequence is released openly for reuse. The *Podarcis erhardii* genome assembly is provided by the Wellcome Sanger Institute Tree of Life Programme ( https://www.sanger.ac.uk/programme/tree-of-life/) and is part of the Vertebrate Genomes Project (PRJNA489243). All raw sequence data and the assembly have been deposited in INSDC databases. The genome will be annotated using available RNA-Seq data and presented through the Ensembl pipeline at the European Bioinformatics Institute. Raw data and assembly accession identifiers are reported in Table 1 and Table 2.

## Author information

Members of the Wellcome Sanger Institute Tree of Life Management, Samples and Laboratory team are listed here: https://doi.org/10.5281/zenodo.12162482.

Members of Wellcome Sanger Institute Scientific Operations: Sequencing Operations are listed here: https://doi.org/10.5281/zenodo.12165051.

Members of the Wellcome Sanger Institute Tree of Life Core Informatics team are listed here: https://doi.org/10.5281/zenodo.12160324.

Members of the Tree of Life Core Informatics collective are listed here: https://doi.org/10.5281/zenodo.12205391.
