# Supplementary material for: The genome sequence of Erhard’s wall lizard, Podarcis erhardii (Bedriaga, 1882)
Source: Wellcome Open Res. 2025 Apr 23;10:212. [Version 1] doi: 10.12688/wellcomeopenres.24041.1 (PMC12198748; doi:10.12688/wellcomeopenres.24041.1)
Supplement: Peer review history

# Reviewer report: Reviewer response for version 1

**DOI:** 10.21956/wellcomeopenres.26525.r125224

**Published:** 2025-07-02

**Authors:** Merly Escalona

**Affiliations:** 1 University of California, Santa Cruz, California, USA

The authors describe the efforts for genome sequencing and assembly of a female *Podarcis erhardii* lizard. These efforts include a phased nuclear genome assembly, mitochondrial genome assembly, and annotation of both nuclear and mitochondrial assemblies. All the resources generated are of high quality and satisfy the minimum metrics of the Earth BioGenome Project and Vertebrate Genome Project as established by the Rhie et al. 2020 paper and the documentation of the EBP. 

 However, I have 2 minor comments and some formatting notes.

 Minor comments: 

- While both the nuclear and mitochondrial genomes have been assembled, annotated, and made publicly available, there are no metrics for quality description of the mitochondrial genome, and it would be good to share those as well.
- Are the contaminants from Apicomplexa expected on this species? Genus? Is that single contig the full genome of the parasite?

Formatting notes:

- There are some inconsistencies with how locations are described:
  - Section Background - Paragraph 6 - Last line: Andros -> Andros, Greece.
  - Section Methods - Sample acquisition - Paragraph 1 - Line3-4: Andros in the northern Cyclades > Andros in the northern Cyclades, Greece.
  - Section Methods - Nucleic acid extraction - Paragraph 1: WSI DToL > WSI DToL, UK.
  - Section Methods - Hi-C sample preparation and crosslinking
    - Thermo Fisher Scientific > Thermo Fisher Scientific, Massachusetts, USA or Thermo Fisher Scientific, USA.
  - Section Methods - Library preparation - HiC:
    - Covaris > Covaris, Massachusetts, USA or Covaris, USA.
- Is PacBio HiFi header level 3? If so, wouldn’t Hi-C also be formatted the same way?
- Section Genome assembly, curation and evaluation - Assembly quality assessment - Paragraph 3: Is it blobtoolkit or Blobtoolkit or BlobToolkit? Please be consistent.

Are sufficient details of methods and materials provided to allow replication by others?

Yes

Is the rationale for creating the dataset(s) clearly described?

Yes

Are the datasets clearly presented in a useable and accessible format?

Yes

Are the protocols appropriate and is the work technically sound?

Yes

Reviewer Expertise:

genome assembly, bioinformatics

I confirm that I have read this submission and believe that I have an appropriate level of expertise to confirm that it is of an acceptable scientific standard.

# Reviewer report: Reviewer response for version 1

**DOI:** 10.21956/wellcomeopenres.26525.r125225

**Published:** 2025-06-25

**Authors:** Roberto Biello

**Affiliations:** 1 John Innes Centre Department of Crop Genetics (Ringgold ID: 534071), Norwich, England, UK; 2 University of Ferrara, Ferrara, Italy

The manuscript presents a chromosome-level genome assembly of the Erhard's wall lizard ( *Podarcis erhardii*). It represents an important contribution to genomic resources for the *Podarcis* genus and related taxa. Sequencing was performed using PacBio HiFi and Hi-C data, and assemblies were generated for both haplotypes. However, chromosome-level resolution was achieved only for the first haplotype. The assemblies meet high-quality standards, supported by BUSCO scores, k-mer completeness, and a high percentage of sequence mapping to chromosomes. The methods are clearly described, and the associated data have been made publicly available.

 Minor comments:

 - At the end of the introduction, the authors stated that this species was sequenced as part of the DToL project, which focuses on sequencing all eukaryotic species in Britain and Ireland. Since *P. erhardii* is a Balkan species, the authors should better explain why it was included in the DToL project.

 - In the sample acquisition section, the authors mentioned that they collected the tip of the tail and preserved it in ethanol. However, for the Hi-C sample preparation, they reported using 20–50 mg of frozen tissue stored at -80°C. These descriptions are inconsistent, and the authors should clarify it.

 - The rationale for choosing the subspecies *mykonensis* for genome sequencing was not provided.

 - The authors described that PacBio reads are aligned using SAMtools within the BlobToolKit pipeline. In fact, read alignment is performed using minimap2, while SAMtools is used for downstream processing of the SAM/BAM files. This should be corrected in the methods section.

 - The authors stated that Figure 4 illustrates the “completeness of the assembly,” but this is not correct given that analysis shows the cumulative length for all scaffolds.

Are sufficient details of methods and materials provided to allow replication by others?

Yes

Is the rationale for creating the dataset(s) clearly described?

Partly

Are the datasets clearly presented in a useable and accessible format?

Yes

Are the protocols appropriate and is the work technically sound?

Yes

Reviewer Expertise:

Comparative genomics, Genome assembly and annotation, Evolutionary biology, Conservation genomics

I confirm that I have read this submission and believe that I have an appropriate level of expertise to confirm that it is of an acceptable scientific standard.

# Reviewer report: Reviewer response for version 1

**DOI:** 10.21956/wellcomeopenres.26525.r122613

**Published:** 2025-05-10

**Authors:** Erin Westeen

**Affiliations:** 1 University of California Berkeley, Berkeley, California, USA

The authors present a genome sequence of a female individual of Erhard's wall lizard, Podarcis erhardii. The genome was sequenced using PacBio long reads and Hi-C to capture chromosome conformation. The authors assembled two haplotypes and the mitochondrial genome.

 The article is concise and well-written. I have only small suggestions.

 when beginning a sentence with the scientific name, Podarcis should be spelled out (rather than P.).

 In the methods, perhaps it should be mentioned that Andros (and the Cyclades) are in Greece?

Are sufficient details of methods and materials provided to allow replication by others?

Yes

Is the rationale for creating the dataset(s) clearly described?

Yes

Are the datasets clearly presented in a useable and accessible format?

Yes

Are the protocols appropriate and is the work technically sound?

Yes

Reviewer Expertise:

phenotypic evolution, comparative biology, herpetology, genomics

I confirm that I have read this submission and believe that I have an appropriate level of expertise to confirm that it is of an acceptable scientific standard.
